# Preclinical models derived from endoscopic ultrasound-guided tissue acquisition for individualized treatment of pancreatic ductal adenocarcinoma

**DOI:** 10.3389/fmed.2022.934974

**Published:** 2023-01-05

**Authors:** Ting Tong, Chao Zhang, Jingbo Li, Minzi Deng, Xiaoyan Wang

**Affiliations:** ^1^Endoscopic Center, The First Affiliated Hospital of Xiamen University, Xiamen, China; ^2^Endoscopic Center, Department of Gastroenterology, The Third Xiangya Hospital, Central South University, Changsha, China; ^3^Hunan Key Laboratory of Non-Resolving Inflammation and Cancer, Central South University, Changsha, China

**Keywords:** endoscopic ultrasound-guided tissue acquisition, individualized medicine, pancreatic ductal adenocarcinoma, preclinical model, precision medicine

## Abstract

Pancreatic ductal adenocarcinoma (PDAC) is an aggressive malignancy with poor outcomes. Although the management strategies have evolved in recent years, the PDAC 5-year survival rate remains at only 9%; it may become the second leading cause of cancer death in the USA by 2030. Only 15–20% of PDAC patients are eligible to undergo surgery; diagnostic biopsies and individualized treatment present a more significant challenge for the remaining group. Endoscopic ultrasound-guided tissue acquisition (EUS-TA) has been widely used in the diagnosis of pancreatic masses. With the advancement of this sampling technique, adequate specimens can be obtained from all patients with PDAC in both early and late clinical stages. Recent data suggest that the specimens obtained from EUS-TA might be used to establish viable preclinical models, which conserve the genetic mutation and preserve the heterogeneity of the original tumors. Additionally, any drug sensitivity evident in the EUS-TA-derived preclinical models might predict the clinical response, thus guiding the prospective therapeutic selection. As we move toward the era of precision medicine, this review provides an update on the role of EUS-TA as a method for obtaining genetic material used in preclinical models that can assess and stratify individuals according to their individual cancer biology.

## 1. Introduction

Pancreatic cancer (PC) is an aggressive and lethal tumor (1–3). The 5-year survival rate for all stages combined stands at a mere 9% (1, 4, 5). In contrast to the steady increase in survival for most cancer types, the limited improvement for PC is partly because up to four-fifths of cases are diagnosed at an advanced stage (1, 2, 4, 6) Several projections indicate that PC will surpass breast cancer as the second and third leading cause of cancer death in the USA (7–9) and the European Union (10, 11), respectively, by 2030. Pancreatic ductal adenocarcinoma (PDAC) and its variants account for over 90% of PC (7, 12). Surgical resection is the only potentially curative treatment for patients with PDAC (2, 3). However, the majority of patients (80–85%) are diagnosed with locally advanced or metastatic disease, thus becoming ineligible for surgery (13, 14). Additionally, post-operative PDAC recurrence might be observed in up to 80% of the cases (15). The therapeutic options for these patients are limited to combination chemotherapy regimens (1, 3). Gemcitabine/nab-paclitaxel or FOLFIRINOX (5-Fluorouracil, leucovorin, irinotecan, and oxaliplatin) remain the gold standard for PDAC chemotherapy with a significant average increase of survival of up to 1 year (15). Therefore, chemotherapy is essential for the treatment of late-stage PDAC. Many PDAC tumors are chemorefractory owing to an unknown mechanism, yet a smaller subset of patients exhibit a significant response to chemotherapy (6, 16). Reportedly, the objective response rates stand at 31.6% for FOLFIRINOX-treated patients and 10–23% for gemcitabine-treated patients, while 70 and 80–90% of these patients are non-responders, respectively (17, 18). Hence, it is crucial to identify the subgroups sensitive to current chemotherapy drugs and determine individualized treatment options for those resistant to the approved therapeutic regimens.

The term “individualized medicine” or “precision medicine” refers to the delivery of custom-designed treatments to patients (19). The goal of precision medicine should be to cure cancer, or at least to increase the overall disease-free and progression-free survival rates (20). In contrast to conventional therapy for a specific pathological type of cancer, individualized medicine considers individual patient differences and stratifies patients accordingly (21). This would optimize the effectiveness of the treatment and contribute to avoiding the side effects of ineffective therapies. Given the low response rate to current chemotherapy regimens, precision medicine has a good application prospect in PDAC treatment. However, since few patients are eligible to undergo surgical resection of the lesions, it is difficult for most patients to obtain adequate tumor specimens. Therefore, the widespread application of precision medicine is limited. Besides, studies based on surgical specimens alone, such as genetic analyses, drug response analyses, and multi-omics analyses, among others, may be biased due to the early and middle clinical stages of patients, affecting further application.

Endoscopic ultrasound-guided tissue acquisition (EUS-TA) now is a widely used method to obtain specimens and diagnoses of PDAC. Endoscopic ultrasound (EUS), which is combination of B-mode and color Doppler EUS imaging, operating at 7.5 MHz, could not only delineate tumor location and size, but also presents a good accuracy for detecting lymph node metastasis, vascular invasion and predicting resectability (8). Particularly, when combined with tissue acquisition, i.e., endoscopic ultrasound-guided tissue acquisition (EUS-TA), the pretreatment diagnostic yields and staging accuracy have been increased (8, 22–24). Recent data suggest that EUS-TA, including endoscopic ultrasound-guided fine-needle aspiration [EUS-FNA; including EchoTip Ultra (Cook Medical), EZ Shot 3 Plus (Olympus Medical Systems), etc.] (25, 26) and endoscopic ultrasound-guided fine-needle biopsy [EUS-FNB; including EchoTip ProCore (Cook Medical), SharkCore (Medtronic), Acquire (Boston Scientific), Sono Tip Topgain (MediGlobe), etc.] (25–28), could obtain adequate specimens for diagnosis and personalized treatment (27, 29–31), with a sensitivity of 86.3–98.4% and a specificity of 100% for the diagnosis of pancreatic malignancy (32). Furthermore, EUS-TA is generally considered a safe procedure with low morbidity (0.59–0.98%) and mortality (0.02%) rates (6, 33). To overcome the clinical challenge of recruiting more patients for subsequent individualized treatment, many studies use EUS-TA as an alternative to procure biological material (2, 34–36).

In general, precision medicine for cancer includes two key aspects: Genetic profiling and drug target validation through patient-derived models (37). The former aspect has been studied and reviewed in depth (27, 38, 39). Here we review the advances in establishing and applying different patient-derived preclinical models of PDAC isolated from EUS-TA specimens for drug target validation.

## 2. Characteristics of EUS-TA specimens for establishing patient-derived preclinical models

### 2.1. Characteristics of EUS-TA specimens compared to surgical specimens

The differences between specimens obtained from EUS-TA and surgery are mainly reflected in the following three aspects. First, compared to surgical specimens, EUS-TA specimens can be obtained at any stage of the disease, and the procedure is easier to repeat than other biopsy techniques. Furthermore, EUS-TA can be used to obtain specimens from patients with PDAC before administering neoadjuvant chemotherapy, since neoadjuvant chemotherapy may reduce the number of viable tumor cells in a sample, leading to a 40% drop in engraftment rates (40, 41). Additionally, surgical specimens contain large amounts of stromal tissue, while EUS-TA samples contain a higher proportion of cancer cells but typically contain blood, inflammatory cells, and even gastrointestinal wall epithelial cells (6). Therefore, it was postulated that the differences seen in the transcriptome profile from EUS-TA and resection specimens may reflect differences in the nature and degree of “contaminating” cells rather than differences in the tumor profile (6).

Although the specimens obtained by different methods have their own characteristics, the genomes they contain do not differ significantly. Several studies showed that the gene mutations of most EUS-FNA and EUS-FNB specimens were similar to those of surgical specimens (6, 42), revealing that EUS-TA samples can retain the genetic signature of the original tumor and be used for individualized treatment and further research. Besides, EUS-FNB has the potential for superior performance compared to that of EUS-FNA in obtaining specimens for precision medicine, especially when the evaluated lesion was small (42).

### 2.2. Number of passes of EUS-TA needed for establishing preclinical models

The amount of the specimens is crucial for model establishment. However, different studies have come to different conclusions regarding the amount of specimens required for modeling, and there is not uniform yet. Since there is no study report on the number of cells, grams, milliliters, or size required to successfully culture the preclinical model, we used the number of passes as a criterion to assess the amount of samples required to culture the preclinical model. The study of Lee et al. showed that there was no statistically significant difference (87.5 vs. 100%, *P* = 0.58) between two passes and three passes in acquiring the histological core when used a 20-gauge (G) FNB needle (EchoTip ProCore™ Endoscopic Ultrasound Needle, Cook Medical Inc., Bloomington, IN, USA), both of which could successfully establish the patient-derived cell (PDC) model (43). In another study, the endoscopists determined the number of passes according to the results of macroscopic on-site evaluation (MOSE), and compared the different success rates of PDAC organoid isolation (P0) and establishment (P5) in cell culture from a single FNB (22-gauge, SharkCore FNB Exchange System, Medtronic Inc., Minneapolis, MN, USA; or Acquire FNB Device, Boston Scientific Inc., Marlborough, MA, USA) pass and a double FNB pass. The results showed that there was no statistical significance (P0: *P* = 0.5175; P5: *P* = 0.3287) between the two groups. However, the success rates of organoid isolation (P0) and proliferation from P0 to P5 were slightly higher in single-pass biopsies [P0: 88% (22/25); P5: 81% (34/42)] than in double-pass biopsies [P0: 76% (19/25); P5: 60% (25/42)], respectively (44). This might be explained by the learning curve effect with organoid creation in the laboratory, as the single-pass specimens were obtained further along within this study protocol. Additionally, two patients developed acute pancreatitis, and two patients experienced bleeding at the FNB site after the EUS-FNB procedure in the double-pass cohort (44), suggesting that two passes are not superior to a single pass for the model establishment and they may increase the risk of adverse events.

Both of the FNA and FNB are used in obtaining specimens for preclinical model creation, but there is no comparison revealed which one is more suitable. In spite of this, EUS-FNB has been reported to require fewer passes to achieve a conclusive diagnosis, and immunohistochemistry was more successfully performed in FNB samples (31, 45, 46). Additionally, some studies comparing whether the two types of needle can obtain adequate specimen for genomic profiling, yield of DNA, and theranostic potential have revealed that EUS-FNB were considerably superior to EUS-FNA in these aspects (27, 47). Moreover, FNB should be given priority when tumors are ≤ 3 cm or for tumors located in the head/neck of the pancreas (47). Hence, the FNB needle is expected to perform better in obtaining tissue for establishing preclinical models. However, the question of which is better, FNA or FNB, and whether the application of rapid on-site evaluation (ROSE), MOSE, fanning, wet suction technique, or other techniques, will improve the culture success rate, still needs to be confirmed by high-quality clinical trials.

### 2.3. Processing method of EUS-TA specimens and the medium of preclinical models *in vitro*

Unlike the surgical specimens obtained as large cell blocks, EUS-TA specimens are recovered as strips or fragments. Because of this, EUS-TA specimens can be directly used for cell isolation and culture regardless of the use of enzymatic digestion. Several studies showed that FNA samples were directly mixed with 100 mL of Matrigel and injected in the upper right flank of a nude mouse to establish patient-derived xenografts (PDXs) (36, 48, 49), or embedded in Matrigel and overlaid with a basal culture media with several niche factors to create patient-derived organoids (PDOs) (5, 50). Although there is no study to compare the effects of digestion versus non-digestion of TA specimens for the establishment of PDAC preclinical models, one study has assessed the impact of digestion on immune cells (51). Vilgelm et al. demonstrated that despite the same initial cell plating density, the immune cell survival in FNA-PDOs (without digestion) culture was significantly higher than digestion-based PDOs at 2 weeks (*p* < 0.05) (51). Besides, the study indicated that FNA, a gentler extraction technique, might be beneficial for immune cell survival (51). Additionally, because EUS-TA specimens contain more blood than surgical specimens, red blood cells need to be lysed with Red Blood Cell Lysis Buffer before culturing (5, 50).

Appropriate niche factors are important for the establishment and passage of *in vitro* models and for screening cancer cells. Table 1 lists the medium, including the niche factors, used in the previous studies for culturing PDCs and PDOs derived from EUS-TA samples.

**TABLE 1:** Culture medium and co-culture cells of patient-derived cells (PDCs) and patient-derived organoids (PDOs) derived from pancreatic ductal adenocarcinoma (PDAC) specimens obtained by endoscopic ultrasound-guided tissue acquisition (EUS-TA).

References	Culture medium	Co-culture cells
**PDCs**		
Lee et al. (43)	Rho-kinase inhibitor (Y-27632). The rest were not detailed.	J2 mouse fibroblasts after lethal irradiation.
Lee et al. (34)	Ham’s F-12 nutrient mix (70%), complete DMEM (25%), hydrocortisone (0.4 mg/mL), insulin (5 mg/mL), 8.4 ng/mL cholera toxin (8.4 ng/mL), EGF (10 ng/mL), FBS (5%), adenine (24 mg/mL), gentamycin (10 mg/mL), Amphotericin B (250 ng/mL), Y-27632 (5 mM).	J2 mouse fibroblasts after lethal irradiation.
**PDOs**		
Tiriac et al. (2)	Advanced DMEM/F12, HEPES 10 mM, Glutamax (1X), A83-01 (500 nM), hEGF (50 ng/mL), mNoggin (100 ng/mL), hFGF10 (100 ng/mL), hGastrin I (0.01 μM), N-acetylcysteine (1.25 mM), Nicotinamide (10 mM), PGE2 (1 μM), B27 (1X), R-spondin1 conditioned media (10%), Afamin/Wnt3A conditioned media (50%).	NM
Juiz et al. (82)	Advanced DMEM/F12, HEPES (10 mM), Glutamax (1X), penicillin/streptomycin, animal-free recombinant hFGF10 (100 ng/mL), animal-free recombinant hEGF (50 ng/mL), recombinant hNoggin (100 ng/mL), Wnt3a-conditioned medium (30%), RSPO1-conditioned medium (10%), hGastrin 1 (10 nM), Nicotinamide (10 mM), N acetylcysteine (1.25 mM), B27 (1x), A83-01 (500 nM), Y27632 (10.5 μM).	NM
Armstrong et al. (88)	Advanced DMEM/F12, HEPES (1 M), B27 (1X), N2 (1X), N-Acetylcysteine (1 mM), nicotinamide (10 mM), hGastrin (0.1 mol/L), hEGF (50 ng/mL), A83-01 (500 nM), Y-27632, hFGF-10 (100 ng/mL), Wnt3A-R-spondin1-Noggin condition media (50%).	NM
Lee, et al. (53)	Advanced DMEM/F12, Gluta MAX (2 mM), HEPES (10 mM), penicillin (100 U/mL), hygromycin B (100 U/mL), streptomycin (100 μg/mL), Wnt-3A (50%), R-spondin 1 (50%), mNoggin conditioned medium (50%), hEGF (50 ng/mL), hFGF-10 (100 ng/mL), nicotinamide (10 mM), A83-01 (500 nM), B27 (1X), N-acetylcysteine (1.25 mM), FBS (10%), hGastrin I (0.01 μM).	NM
Boj et al. (80)	Advanced DMEM/F12, HEPES (1X), Glutamax (1X), penicillin/streptomycin (1X), B27 (1X), Primocin (1 mg/ml), N-acetyl-L-cysteine (1 mM), Wnt3a-conditioned medium (50%), RSPO1-conditioned medium (10%), Noggin-conditioned medium (10%) or recombinant protein (0.1 mg/ml), EGF (50 ng/ml), Gastrin (10 nM), FGF-10 (100 ng/ml), Nicotinamide (10 mM), A83-01 (0.5 mM).	NM
Tiriac et al. (3)	Advanced DMEM/F12, HEPES (10 mmol/L), Glutamax (1X), A83-01 (500 nmol/L), hEGF (50 ng/mL), mNoggin (100 ng/mL), hFGF10 (100 ng/mL), hGastrin I (0.01 μmol/L), N-acetylcysteine (1.25 mmol/L), nicotinamide (10 mmol/L), PGE2 (1 μmol/L), B27 (1X), R-spondin1 conditioned media (10%), and afamin/Wnt3A conditioned media (50%).	NM
Seino et al. (5)	Advanced DMEM/F12, penicillin/streptomycin, HEPES (10 mM), GlutaMAX (2 mM), B27 (1X), Gastrin I (10 nM), N-acetylcysteine (1 mM), recombinant mEGF (50 ng/ml), recombinant mNoggin (100 ng/ml), R-spondin-1 conditioned medium (10%), Afamin-Wnt-3A serum-free conditioned medium (25%), A83-01 (500 nM), SB202190 (10 mM).	CAFs
Bian et al. (50)	Advanced DMEM/F12, HEPES (10 mM), Glutamax (1X), penicillin/streptomycin, Animal-Free Recombinant Human FGF10 (100 ng/ml), Animal-Free Recombinant hEGF (50 ng/ml), Recombinant hNoggin (100 ng/ml), Wnt3a-conditioned medium (30%), RSPO1-conditioned medium (10%), hGastrin 1 (10 nM), Nicotinamide (10 mM), N acetylcysteine (1.25 mM), B27 (1x), A83-01 (500 nM), Y27632 (10.5 μM).	NM
Hennig et al. (83)	Advanced DMEM/F12, Wnt3a-conditioned medium (50%), noggin conditioned medium (10%), RSPO1-conditioned medium (10%), B27 (1X), nicotinamide (10 mM), gastrin (1 nM), N-acetyl-Lcysteine (1 mM), primocin (1 mg/ml), recombinant mEGF (50 ng/ml), recombinant hFGF10 (100 ng/ml), A83-01 (0.5 μM), N2 (1X).	NM
Dantes et al. (35)	Advanced DMEM/F12, HEPES (10 mM), GlutaMax (1X), B27 (1X), Primocin (100 μg/mL), N-acetyl-L-cysteine (1.25 mM), recombinant hWnt3a protein (100 ng/mL) or Wnt3a-conditioned medium (50%), RSPO1-conditioned medium (10%) or recombinant hR-Spondin 1 protein (500 ng/mL), mNoggin (100 ng/mL), EGF (50 ng/mL), Gastrin (10 nM), FGF10 (100 ng/mL), Nicotinamide (10 mM), Y-27632 (10 μM), A83-01 (0.5 μM).	NM

PDC, patient-derived cell; PDO, patient-derived organoid; PDAC, pancreatic ductal adenocarcinoma; EUS-TA, endoscopic ultrasound-guided tissue acquisition; DMEM, Dulbecco’s modified Eagle’s medium; EGF, epidermal growth factor; FGF, fetal growth factor; FBS, fetal bovine serum; CAF, cancer-associated fibroblasts; NM, not mentioned.

### 2.4. Success rate of establishing patient-derived preclinical models

In different models, the success rate in establishing patient-derived preclinical models ranges from 36.4 to 87% (Table 2). Previously, Lee et al. showed that the establishment rate of PDC was 36.4% (8/22) using the FNB samples (43). Hermans et al. showed that the engraftment rate of FNB samples (6/10, 60%) was lower than that of surgical samples (4/4, 100%). However, it seemed that FNB-derived PDXs needed a shorter time to tumor formation at F1 (17.2 vs. 19 weeks; *P* = 0.67); the period of time became statistically difference at F3 (6.3 vs. 11.3 weeks; *P* = 0.02) when compared to surgery-derived PDXs (52). In 2018, a clinical trial was conducted to evaluate the feasibility of creating human PDAC organoids by EUS-FNB (2, 44). After obtaining sufficient samples to reach a diagnosis, one to two additional passes were performed for organoid creation. Successful creation of organoids (P0) was achieved in 87% (33/38) tumors, and 66% (25/38) organoids could grow ≥ 5 passages, demonstrating that EUS-FNB can successfully and rapidly create pancreatic cancer organoids at the time of initial diagnosis (2). The failure to reach P5 in some specimens is likely due to the organoid medium lacking the growth factors when a high number of normal epithelial cells are present (44).

**TABLE 2:** Data on the establishment of preclinical models for pancreatic ductal adenocarcinoma (PDAC) patients using endoscopic ultrasound-guided tissue acquisition (EUS-TA) alone and combined with other approaches as well as the accuracy of drug screening results.

References	Tissue acquisition approaches	Time to model formation	Number of models created	Number of passages	Correlation between drug screening results and clinical effect
**PDCs**					
Lee et al. (43)	FNB (20 G, 2–3 passes)	7–14 d	8/22 (36.4%)	>20	NM
Lee et al. (34)	FNB; Surgery; Percutaneous biopsy	NM	FNB: 15; Surgery: 12; Percutaneous biopsy: 1	NM	The IC50 value of each drug was statistically lower in the responder group than in the non-responder group.
PDXs					
Berry et al. (67)	FNA (22 G, 1 pass)	3–6 m (1,000 mm^3^)	2	NM	NM
Duconseil et al. (48)	FNA; Surgery	2–6 m (1,000 mm^3^)	FNA: 6; Surgery: 11	≥6	NM
Gayet et al. (68)	FNA; Surgery	NM	Total: 17	NM	NM
Hermans et al. (52)	FNB (22 G); Surgery	Passage 1: 8–27 d/Passage 3: 5–8 d (1,000–1,500 mm^3^)	FNB: 6/10 (60%); Surgery: 4/4 (100%)	5	NM
Allaway et al. (62)	FNA (1–2 passes); Surgery	18 w (5 mm^3^)	FNA: 9/24 (37.5%); Surgery: 10/10 (100%)	5	NM
Barraud et al. (69)	FNA; Surgery	NM	Total: 23	NM	NM
Nicolle et al. (36)	FNA; Surgery	NM	Total: 30	NM	NM
Bian et al. (49)	FNA; Surgery	2–6 m (1,000 mm^3^)	FNA: 25; Surgery: 30	≥6	NM
PDOs					
Tiriac et al. (2)	FNB (22 G, 1–2 passes)	2 w (P0)	33/38 (87%)	5	NM
Juiz et al. (82)	FNB	NM	20	NM	NM
Armstrong et al. (88)	FNB	NM	15/18 (83.33%)	NM	NM
Lee, et al. (53)	FNA (19G or 20G, 1 pass)	NM	P0: 14/20 (70%); P5: 12/20 (60%)	≥5	The moderate tendency of correlation was found between the organoid’s drug response and the patient’s OS (Spearman correlation coefficient, ρOS = 0.48).
Boj et al. (80)	FNA; Surgery	NM	FNA: 2; Surgery: NM	Indefinitely	NM
Tiriac et al. (3)	FNB; Surgery; Rapid autopsy; VATSR	NM	FNB: 43/60 (72%); Surgery: 61/78 (78%); Rapid autopsy + VATSR: 10/21 (48%)	≥5	The clinical results showed that 5, 1, and 2 patients were sensitive, moderately sensitive and resistant to the corresponding chemotherapy agents, which was consistent with the drug screening results.
Seino et al. (5)	FNA; Surgery; Aascites; ERCP	NM	FNA: 33; Surgery: 12; Aascites: 3; ERCP: 1	NM	NM
Bian et al. (50)	FNA; Surgery	2–3 w	FNA: 85%; Surgery: NM	NM	NM
Hennig et al. (83)	FNA; Surgery	NM	FNA: 5/6 (83%); Surgery: 17/25 (68%)	>10	NM
Dantes et al. (35)	FNA (19/20/22 G, 1 pass); Surgery	NM	FNA: 6; Surgery: 4	≥5	NM

PDAC, pancreatic ductal adenocarcinoma; EUS-TA, endoscopic ultrasound-guided tissue acquisition; PDC, patient-derived cell; FNB, fine-needle biopsy; G, gauge; d, days; NM, not mentioned; PDX, patient-derived xenograft; FNA, fine-needle aspiration; m, months; w, weeks; PDO, patient-derived organoid; VATSR, video-assisted thoracoscopic surgical resection; ERCP, endoscopic retrograde cholangiopancreatography.

Studies have also evaluated the utility of collecting tissues of patients with PDAC using EUS-FNA to create organoids. The results of Lee and colleagues’ study showed a 70% success rate for PDO isolation (14/20) and 60% (12/20) for PDO growing more than 5 passages (53). In addition, for EUS-FNA, a success rate of between 62 and 100% for PDAC original tumors and up to 70% for PDAC liver metastases was previously reported in another study (15).

## 3. Application of different types of EUS-TA specimen-derived PDAC preclinical models in precision medicine

### 3.1. EUS-TA specimen-derived PDC models

Patient-derived cell (PDC) plays a vital role in precision medicine. It is a type of 2D cell, which is generally easy to culture, propagate, cryopreserve, and manipulate both genetically and chemically, and the cost is relatively low (34, 54, 55). Recently, a novel type of PDC, named conditionally reprogrammed cells (CRCs), has been reported. Researchers co-cultured PDC with J2 murine fibroblast feeder cells and a medium containing the Rho-kinase inhibitor (Y-27632) and proved that PDC could be constructed using a small piece of tumor tissue obtained from EUS-FNB (34, 43). The CRC cultures can be passaged for long periods without genomic alterations and could maintain the heterogeneity of cells present in a biopsy and make high throughput drug screening possible owing to their rapid expansion (4–6 weeks) (34, 56, 57). Additionally, there is no need for Matrigel, an extracellular matrix that may interfere with drug penetration or cause adverse drug screening results (34). A study tested drug sensitivity using CRCs obtained from EUS-TA, revealing that the IC50 value of each drug was statistically lower in the responder group than in the non-responder group (34; Table 2). Therefore, by evaluating the drug sensitivity of a large panel of clinical agents, the EUS-TA specimen-derived PDC (TA-PDCs) platform might identify the new drugs useful as therapeutic options for individual patients.

Despite the advantages listed above, TA-PDCs also have some limitations as PDC derived from other approaches, including surgery (Table 3). First, compared to PDXs, the monolayer cells lack the gradients and extracellular matrix scaffold. Cell-cell contact and cellular polarity are difficult to model in this setting, and cancer cells lack the structural organization and functional differentiation present *in vivo* (34, 54, 58, 59). The tumor microenvironment is not a bystander but rather an active participant in tumor progression (60), and evaluating the impact of the tumor microenvironment, such as stromal cells, on tumor cell growth is much more challenging (34). Recent studies reported organoid culture after CRC establishment, which can be a solution to this problem in cancer research (34, 61). Additionally, some TA-PDCs derived from malignant tumors were often non-malignant, without tumor derived mutations. The growth factor consumption of non-malignant cells may impact the proliferation of tumor cells (44). Detecting the PDAC markers by PCR aimed at detecting a common gene such *KRAS*, performing tumor formation assays, and performing targeted sequencing to identify tumor cells at the start may resolve this situation. Despite the above limitations, TA-derived PDC can provide helpful information for personalized treatments *via* drug screening or toxicity tests.

**TABLE 3:** The characteristics of preclinical models for human pancreatic ductal adenocarcinoma (PDAC) derived from endoscopic ultrasound-guided tissue acquisition (EUS-TA).

Model type	Advantages	Disadvantages
TA-PDCs	① Can be constructed with a small piece of tumor tissue. ② Rapid generation of model. ③ Retain genetic characteristics and heterogeneity of tumor. ④ Easy to culture, propagate, cryopreserve, and manipulate. ⑤ Suitable for high throughput drug screening. ⑥ Longitudinal assessment of chemosensitivity. ⑦ Relative low cost.	① Absence of gradients, extracellular matrix scaffold, and tumor microenvironment. ② Difficult to model cell-cell contact. ③ Lack the structural organization and functional differentiation.
TA-PDXs	① Can be constructed with a small piece of tumor tissue. ② Retain genetic characteristics and heterogeneity of tumor. ③ Model the cross-talk between cancer cells and stromal components. ④ Separately analyze the human grafted cancerous and infiltrating mouse stromal cells. ⑤ Longitudinal assessment of chemosensitivity.	① Expensive. ② Low survival rate of immunocompromised mice. ③ Time delay to engraftment and drug screening. ④ Suitable for low throughput drug screening. ⑤ Not suitable to model the interactions between cancer cells and immune cells.
TA-PDOs	① Can be constructed with a small piece of tumor tissue. ② Rapid generation of model. ③ Retain genetic characteristics and heterogeneity of tumor. ④ Relatively easy to culture, propagate, cryopreserve, and manipulate. ⑤ Longitudinal assessment of chemosensitivity. ⑥ Suitable for high throughput drug screening. ⑦ Model the interactions between cancer cells and stromal components when co-culture with stromal cells. ⑧ Explore the immunotherapy when co-culture with immune cells.	① Expensive. ② Relatively immature technique.

PDAC, pancreatic ductal adenocarcinoma; EUS-TA, endoscopic ultrasound-guided tissue acquisition; TA, tissue acquisition; PDC, patient-derived cell; PDX, patient-derived xenograft; PDO, patient-derived organoid.

### 3.2. EUS-TA specimen-derived PDX models

As the *in vivo* counterpart to cell lines, PDX tumor models can also retain the patient’s genome and have been widely used. The data from some researches showed that PDX models could be constructed from EUS-TA samples, which are obtained from both primary and metastatic lesions of PDAC (62). EUS-TA specimen-derived PDX (TA-PDX) models can capture the clones with metastatic potential present within the primary tumor, provide a platform for comparing genome-driven therapies before recurrence, and identify potential therapies (62). Additionally, the ability to engraft was negatively correlated with disease-free survival time of patients and could serve as a predictor of the patients’ disease-free survival (63–65). Hermans et al. selected two FNB-PDXs respectively from poorly and moderately differentiated tumors (52, 66). They observed that the growth-rate of the poorly differentiated tumor was higher than that of the moderately differentiated tumor, and poor tumor differentiation exhibited a clear association with epithelial-mesenchymal transition (EMT). Although gemcitabine treatment could reduce the tumor volume and proliferation, it also increased EMT and enhanced the ability of cancer cells to metastasize (66). In contrast to PDC, PDX could model the cross-talk between stromal components and epithelial tumor cells, while maintaining a high genetic stability and preserving the molecular and cellular heterogeneity of the primary tumor (61).

Indeed, each TA-PDX can be molecularly analyzed and concomitantly used for testing biological hypotheses or putative therapeutic targets derived from these analyses. Some studies used TA-samples for sequencing and stratified patients according to molecular signatures, such as a *KRAS* wild type/mutation and *c-MYC* low/high, using the TA-samples to establish PDX cohort according to this stratification. Then, corresponding target inhibitors were applied to treat the PDXs. The results showed that the growth of *KRAS* wild-type and *c-MYC* high PDXs was selectively inhibited by EGFR inhibitors and BET inhibitors, respectively (49, 67). Besides, TA-PDXs were used to profile the PDAC, confirming that there was no relationship between the consensus multi-omics classification and genomic alterations. The most likely explanation was that genetic mutations, amplifications, and deletions were involved in the transformation process of PDAC, whereas the clinical outcome, response to treatments, and the phenotype of the tumors were controlled at the epigenetic level (36). It revealed that the multi-omics analysis is a rich source of novel and reliable therapeutic targets for treating patients with PDAC (36). Through the transcriptomic analysis, the heterogeneity in the RNA expression profile of tumors could be observed, discriminating between potential short-and long-term survivors, and predicting the sensitivity to a set of anticancer drugs (49, 68, 69).

One of the features of the TA-PDX models is that the tumor cell architecture is maintained while the murine stroma replaces human stroma during their construction and passaging, making it a growing chimeric tumor, which better reflects the properties of the original human tumors (36, 60, 66). Sequencing profiles of a mix of human grafted cancerous and infiltrating mouse stromal cells can be analyzed separately *in silico* by unambiguously assigning each sequence to the human or mouse genome (70). With the advent of sequencing-based transcriptomic profiling, TA-PDX offers an ideal setting to study the interactions between the tumor and stromal cells. These works reveal that TA-PDX is a suitable model for preclinical studies, representing the diversity of the primary cancers in which the stroma is reconstituted.

Despite the TA-PDX has the ability to adequately model the *in vivo* condition, it is not perfect (Table 3). First, time is a critical factor in personalized medicine. However, generation of a cohort of PDXs for drug testing might require 2–8 months (55, 71); small biopsy samples may take longer (55). Additionally, waiting for active therapies in relevant TA-PDX, and TA-PDX may be more suitable for low-throughput rather than high-throughput drug screening, which also need extra time (60, 61, 72). Obviously, time-consuming methods are incompatible with the urgency of selecting and implementing treatment regimens for patients because the disease often progresses swiftly. Meanwhile, the generation and maintenance of large numbers of immunocompromised mice to passage the TA-PDXs can be costly (54, 58, 61). Finally, owing to the immunodeficient host, the interactions of cancer cells with the various immune cell types cannot be modeled properly, and it may lead to unforeseeable problems when translating the results of ligand–receptor interactions obtained through this model (55, 73). Nevertheless, Nicolle et al. believed that though the mouse hosts were immunocompromised, the extrathymic maturation such as intestinal T cell differentiation was not precluded. Thus, they believed that TA-PDX tumor models can reproduce partial immune-related phenotype observed in human primary tumors (36). Therefore, whether TA-PDX is appropriate for screening and functional analysis of new immune-therapeutic drugs requires further study.

### 3.3. EUS-TA specimen-derived PDO models

In recent years, based on the successful findings with 3D culture and tumor organoids, a new *ex vivo* preclinical model has been developed and several respective studies have been published. The term refers to a group of cells growing in a 3D structure and using specifically defined media and conditions. It can be generated from primary tissues, metastatic tumors, embryonic stem cells, or pluripotent stem cells (74–76). Generally, 3D spheres and 3D organoids are regarded as the same model, though 3D cultures originate from cell lines in monolayer, and 3D organoids come directly from tissues (58, 77). The current criteria to define a genuine organoid are the following: (1) it recapitulates the identity of the organ it is supposed to model, (2) it mirrors the organ’s cell type diversity, (3) it reproduces the organ-specific functions, and (4) it follows the same self-organization of the tissue it should reproduce (15, 78).

Organoids have been generated using EUS-TA samples with high levels of success in a time frame of just weeks and maintained through indefinite passages while preserving genetic stability, and successfully frozen and thawed, allowing for long-term storage (79–81). It has been widely used in molecular subtyping (50, 82, 83), detection of intratumoral heterogeneity (82, 84, 85), and individualized chemosensitivity testing (50, 53, 83, 86). An EUS-TA specimen-derived PDO (TA-PDO) library or a tumor-chip (incorporating PDOs and stromal cells) could recapitulate the mutational spectrum, transcriptional subtypes and microenvironment of primary PDAC (3, 87). Single-cell transcriptomic analysis showed that a subtype of TA-PDOs could contain more than one phenotype and revealed an unanticipated high heterogeneity of PDAC (82, 84, 85). TA-PDOs also enable longitudinal assessment of chemosensitivity and evaluation of synchronous metastases (3).

Stratification of patients with PDAC is essential to predict their responses to therapies and choose the best treatment due to the heterogeneity of PDAC. Hennig et al. categorized the patients into the established quasimesenchymal, exocrine-like, and classical subtypes based on *KRT81* and *CFTR* immunoreactivity. At the same time, they found that *KRT81*^–^ PDAC organoids tended to be more resistant toward 5-Fluorouracil and oxaliplatin (83). Bian et al. classified patients into two subgroups, *MYC*-high and *MYC*-low, and inhibitors of *c-MYC* transcription were administered in two subgroups. The results showed that these compounds were more efficient in *MYC*-high than in *MYC*-low TA-PDOs (50). Armstrong et al. generated a series of TA-PDOs and used them to screen for sensitivity to 18 compounds. A transcriptomic signature associated with resistance to conventional therapies was identified through RNA sequencing, and it was found that low expression of this “resistance” signature was associated with greater survival in patients with PDAC (88). All the above means that subtyping combined next-generation sequencing using TA-derived PDAC organoids might be beneficial to predict the clinical response. It was reported that PDOs exhibited 100% sensitivity, 93% specificity, 88% positive predictive value, and 100% negative predictive value for the drug response in gastrointestinal cancers (89).

In addition to the above, PDO platforms are also a multipurpose system that can be used to perform a wide spectrum of studies. It can be used for exploring predictive biomarkers of drug response and acquiring specific molecular profiles of patients who may benefit from the tested targeted therapy, which may improve the response rate of patients to targeted or other therapies. Besides, PDOs are also widely used in molecular research. After using cultured PDO for drug screening, Armstrong et al. performed an enrichment analysis to identify pathways based on the genes associated with sensitivity to each of the tested targeted therapies, and showed that multiple growth and signaling pathways (i.e., PI3K, MAPK, Rap1, and Ras) could predicted response to the targeted drugs. For example, the ERBB1 downstream pathway was identified to be associated with sensitivity to ERBB1 inhibitor, erlotinib. The PARP inhibitor olaparib is shown to be associated with multiple pathways of DNA damage repair and chromosome organization (88). Seino et al. used the PDAC TA-PDOs to identify three functional subtypes based on their stem cell niche dependencies on Wnt and R-spondin. The results of their study revealed that niche independency was mainly acquired through driver gene (*KRAS, CDKN2A, TP53, SMAD2*) mutations, whereas the Wnt niche independency was predominantly regulated by epigenetic mechanisms, highlighting a unique niche adaptation process during pancreas tumorigenesis. Their results also provide novel insights into Wnt-based therapeutic strategies against PDACs (5).

Based on the study by Dantes et al., the mutational profile of the primary tumor could be recapitulated by cell-free DNA (cf-DNA) in the TA-PDO supernatant as early as 72 h after the biopsy. This indicated the suitability of this approach to subject TA-PDOs to drug testing in a reduced time frame. This is particularly important for patients for whom biopsy samples were rejected from all genetic testing owing to insufficient tumor quantity (35). Thus, combined with molecular profiling and drug testing, it might facilitate the integration of PDO technology and have a broad implication in clinical practice.

EUS-TA specimen-derived PDOs (TA-PDOs) could be generated not only from neoplastic cells, but also from normal pancreatic ductal cells and pluripotent stem cells after induced differentiation into specific phenotypes (79, 80). These enable PDOs to help study the early stages of disease progression. Based on these characteristics and advantages, TA-PDOs are poised to play an increasingly important role in precision medicine of PDAC, and also can simulate the whole process of tumor development and provide a platform for exploring genetic cooperation.

Notably, though TA-PDOs are attractive for their potential, they still have some limitations (Table 3). One is the high cost. The culture setting of TA-PDOs is complicated, and TA-PDOs need more supplements than primary cells to propagate. Second, similar to PDC, the TA-PDO lack stromal cells present *in vivo*, including cancer-associated fibroblasts, extracellular matrix, pancreatic stellate cells, endothelial cells, immune cells, and various growth factors (15), which represent up to 90% of the tumor volume. The tumor-infiltrating lymphocytes are initially retained within PDOs cultures, which enables to study endogenous immune tumor micro-environmental cells at the beginning. However, the immune cells will be lost over culture propagation (90), and all of these lacked stromal cells and microenvironmental components are required for PDO to closely model PDAC *in vivo*. To surmount this limitation, several groups developed a 3D co-culture system with organoids, fibroblasts, and immune cells to model the interaction between cancer cells and the most abundant cellular components of the tumor microenvironment (87, 91). Through the co-culture model, Öhlund et al. observed an increase in the proliferative rate of organoids and fibroblasts and found heterogeneity between cancer-associated fibroblasts. These findings reflect the complexity of the stroma and its influence on epithelial tumorigenesis (91). Finally, in principle, TA-PDOs are suitable for high throughput screening, but the technical constraints and extensive manipulation have hampered progress toward simple clinical applications (38). Further development is needed to make it more adaptable to high-throughput screening.

## 4. Conclusion and perspectives

Endoscopic ultrasound-guided tissue acquisition (EUS-TA), as a candidate or even a substitute for surgery, is competent in obtaining specimens from patients with all clinical stages of chemo-naive PDAC and providing biological material for genetic analysis, molecular research, stratification, as well as preclinical models establishment, including PDC, PDX, and PDO. In recent years, a variety of TA-derived human PDAC preclinical models have been used for basic and translational studies, which have helped to generate a holistic view of the genetic features of this disease. However, much effort is still needed to optimize the preclinical models derived from EUS-TA, including its establishment and application. First, the sampling and culture protocol needs to be improved and standardized, such as the needed number of passes or length of core tissues, most suitable needle type and gauge, the generation steps of different models, the needed supplements, and culture-supporting matrices, to improve the success rate of model construction, simplify complex manipulation, and reduce the time and cost. Second, different models should be applied according to their different advantages, limitations, and research purposes. For example, TA-PDC, has a relatively fast reproduction, low culture difficulty and cost, is suitable for high-throughput drug screening, and can also be used for the expansion of tumor cells when the initial tumor cells are insufficient, and then used for genetic testing and/or creation of other models (35, 92). TA-PDX, which could simulate the tumor microenvironment present *in vivo*, could be used to study cross-talk between different tumor cells as well as tumor cells and stromal cells. TA-PDO can be propagated in just weeks and obtain accurate drug screening results through high-throughput screening. When co-culturing with immune cells, TA-PDO can also be used to explore the immunotherapy of PDAC. Additionally, when the above three models are combined with sequencing, omics data, or molecular study, they can help identify gene signatures associated with response to novel therapies, stratify patients, and achieve individualized treatment.

**FIGURE 1:**
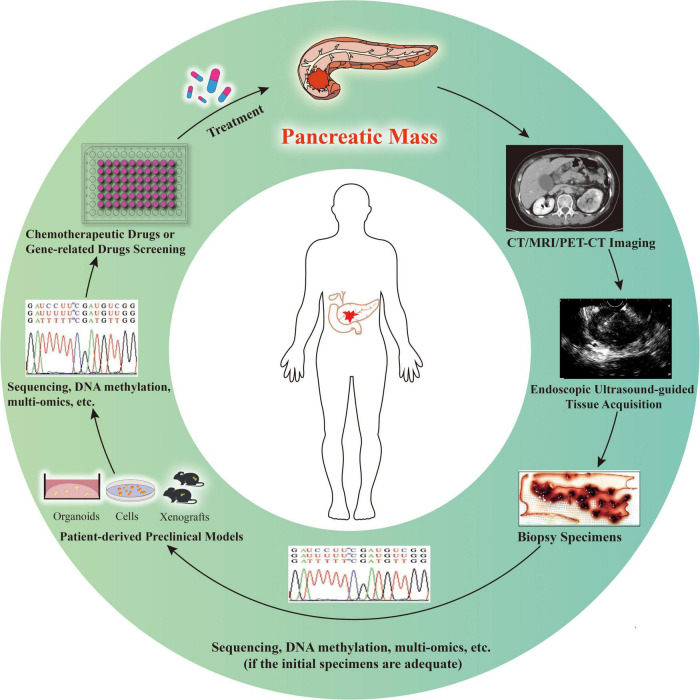
Patient-derived preclinical models for individualized cancer therapy. In the case of imaging highly suspect of pancreatic malignancy, endoscopic ultrasound-guided tissue acquisition is performed to achieve diagnosis and obtain specimens to determine individualized treatment options. If the specimens are adequate for sequencing, DNA methylation, multi-omics, or other molecular studies, as well as construction of preclinical models, studies and model construction can be conducted simultaneously (relatively rare). Otherwise, the models are established first and then various studies are conducted (relatively common). The patient-derived preclinical models might be used for drug screening and/or verification, as well as diverse studies. With the progress of individualized treatment, clinicians will decide whether longitudinal sampling is needed and whether the above process needs to be repeated according to the treatment effect of patients.

Collectively, in the era of individualized treatment, EUS-TA specimen-derived PDAC preclinical models will certainly bring substantial changes to medicine. However, precision medicine for PDAC is still challenging owing to the short median survival of patients with advanced stage disease (3). Before using the models to guide treatment, it is important to determine the successful matching between preclinical models derived from EUS-TA specimens and original tumors and between the drug screening results obtained by the EUS-TA specimen-derived preclinical models and the clinical effects. New high-quality clinical trials and research on individualized PDAC treatment should strive to solve the existing limitations (Table 3). Researchers should also pay attention to the ethical constraints associated with the development of cancer preclinical models. It is expected that routine preparation and application of preclinical models derived from EUS-TA will be a big step in precision medicine and treatment of the disease and benefit more patients with PDAC (Figure 1).

## Author contributions

TT: conceptualization, searching the literature and analyzing the data, and writing—original draft preparation and revision. CZ: writing—original draft preparation. JL: searching the literature and analyzing the data. MD and XW: conceptualization and writing—review and revision. XW: funding acquisition. All authors read and agreed to the published version of the manuscript.
